# Understanding urban-rural inequality in early maternal discharge following facility-based vaginal delivery in Nigeria: A multivariate decomposition analysis

**DOI:** 10.1371/journal.pgph.0005614

**Published:** 2025-12-05

**Authors:** Victor A. Ochagu, Oluwakemi Christie Ogidan, Michael Ekholuenetale

**Affiliations:** 1 Western Regional Coordination Centre, Africa Centre for Disease Control, Abuja, Nigeria; 2 Department of Nursing Science, College of Medicine, Ekiti State University, Ado Ekiti, Ekiti State, Nigeria; 3 Department of Epidemiology and Medical Statistics, Faculty of Public Health, College of Medicine, University of Ibadan, Ibadan, Nigeria; Yale University, UNITED STATES OF AMERICA

## Abstract

The objective of this study was to estimate the urban-rural gap in early maternal discharge following facility-based delivery in Nigeria. A sample of 8614 women aged 15–49 years with national representativeness was extracted and analysed. Multivariate decomposition analysis and concentration index were used to examine the prevalence and urban-rural inequality in early maternal discharge after facility-based vaginal delivery. Approximately 65.6% of rural women and 50.5% of urban dwellers reported early maternal discharge. There was pro-poor distribution of early maternal discharge. The concentration index for rural residence was -0.0817 (SE = 0.0063; p < 0.001) and was -0.0346 (SE = 0.0083; p < 0.001) for urban residence. About 17.6% of early maternal discharge gap was explained by the differences in distributions of characteristics (endowments) between urban and rural residence, while 82.4% of early maternal discharge gap was due to the differences in the distribution of unexplained factors between urban and rural residence. In the multivariate decomposition analysis, having multiple birth, secondary or higher educational levels, moderate or high decision making power, and being from South South geographical region narrowed the urban-rural gap in early maternal discharge. On the other hand, delivery at a private health facility, having 5 + members of household, being from North East, South East and South West geographical region respectively, widened the urban-rural gap in early maternal discharge. The urban-rural gap widened by the disproportionate distribution of women with higher educational level, use of private health facility, large household size and residence in regions like the North East, South East, and South West. These patterns underscore the importance of context-specific interventions that address both structural and individual-level drivers, to ensure equitable postnatal care regardless of geographic or sociodemographic background.

## Introduction

Maternal morbidity and mortality remain critical global health concerns, reaching their highest levels within the first 24hours postpartum and remaining significantly elevated in the days following delivery [1,2]. This period is not only perilous for mothers (with account of 48.9% death occurring in the first 24hours postpartum) [2–5], but also for newborns (with approximately 2.3 million death), as neonatal mortality is similarly concentrated in the immediate days after birth [6]. Despite ongoing efforts to improve maternal and child health [7–11], Nigeria, Africa’s most populous country continues to shoulder a disproportionate burden of maternal and child morbidity and mortality [12]. These adverse outcomes persist as major public health challenges and reflect persistent inequities in healthcare access and service delivery.

Nigeria’s high maternal and child death rates are compounded by complex social, economic, and systemic factors impacting the quality and timeliness of essential health services [13,14]. Facility-based delivery is a cornerstone intervention for reducing preventable deaths; however, merely delivering in a health facility does not guarantee positive outcomes. One crucial aspect of facility care is the timing of maternal discharge following vaginal delivery [15]. The World Health Organization recommends that mothers and newborns remain under skilled care for at least 24hours post-delivery, as this window allows for the detection and management of potential complications such as hemorrhage, infection, or neonatal distress [16]. Early discharge defined as discharge within 24hours of birth can jeopardize the well-being of both mother and child, especially in contexts where follow-up care is limited [17].

A significant urban–rural disparity exists in maternal healthcare utilization and outcomes across Nigeria. Rural women often face greater barriers to accessing skilled care, are more likely to deliver outside of health facilities, and may be discharged earlier when they do access facility-based care [18]. These inequalities are influenced by differences in healthcare infrastructure, provider availability, socioeconomic status, and informational resources between urban and rural settings. However, the underlying contributors to these discharge timing disparities have not been fully elucidated, hindering targeted policy and programmatic responses [19].

This study aims to advance understanding of urban–rural inequalities in early maternal discharge following facility-based vaginal delivery in Nigeria. Using a multivariate decomposition analysis, we disentangle the extent to which observed disparities stem from differences in population characteristics versus differences in the effects of those characteristics. The findings are expected to inform strategies for improving the equity and effectiveness of postpartum care, ultimately contributing to reductions in preventable maternal and child deaths across both urban and rural Nigerian communities.

## Methods

### Ethics statement

This study used publicly available secondary dataset with all identifiers deleted. NDHS received ethical approval from National Health Research Ethics Committee of Nigeria (NHREC) and obtained informed consent from the respondents in accordance with standard ethical procedure. The Health Research Committee assigned number: NHREC/01/01/2007. In addition, ICF institutional Review Board gave ethical approval with ICF Project Number: 132989.0.000.NG.DHS.01. However, the corresponding author of this study was granted permission to use the data, which was gathered in accordance with ethical guidelines, therefore no further participants’ consent was required. Information about DHS guidelines can be found here: http://goo.gl/ny8T6X.

### Data source

Data from the individual woman questionnaire of the 2018 Nigeria Demographic and Health Survey (NDHS) was analysed. The study’s sample consisted of 8614 women aged 15–49 years. The 2018 NDHS was the fifth round carried out by the National Population Commission (NPC). The data was collected from August 14 to December 29, 2018 [20]. The survey employed a stratified, multi-stage cluster design to select the sample, with enumeration areas (EAs) as the sampling units for the first stage. A total of roughly 30 households were selected from the entire list of households in each of the 1,389 EAs that were chosen, with a 99% response rate.

### Sampling technique

The NDHS 2018 employed a three-stage sampling stratification process to ensure that the sample was representative of the general population. This involved first dividing respondents into urban and rural housing strata, then randomly selecting EAs within each stratum, and finally using equal probability sampling to select households within each EA for the survey. The sampling frame for the 2018 NDHS was the Federal Republic of Nigeria’s 2006 Population and Housing Census (NPHC), which was conducted by the National Population Commission. In the 2018 NDHS, the 36 states and the Federal Capital Territory were stratified into urban and rural areas, resulting in 74 distinct sampling strata. Since its start in 1984, the DHS project has been primarily funded by the United States Agency for International Development (USAID) with support from other donors and host countries, and has conducted more than 230 nationally representative and globally comparable household surveys in more than 80 countries. Details of the DHS sampling procedure have been previously published [21].

### Selection and measurements of variables

#### Outcome variables.

Early maternal discharge after facility-based vaginal delivery was investigated. “Time spent at place of delivery” was the question used to measure how long women stayed at a health facility after the child was delivered. Based on WHO classification for early discharge after vaginal birth [22], the outcome variable was coded as “*1* = yes” for women who were discharged in less than 24 hours after vaginal delivery, and “*0 = no*” for those who stayed at least 24 hours, making them to not have experienced early discharge. Women who had facility-based vaginal delivery in the five years prior to the survey were included in this study.

#### Explanatory variables.

The factors examined in this study were based on previous studies [23–28]. birth type: singleton vs. multiple; birth order: First, 2nd/3rd, 4th and above; baby size at birth: large, average, small; baby sex: male vs. female; ANC booking: early vs. late; ANC visits: none, 1–3, 4–7, 8 + ; place of delivery: public health facility vs. private health facility; age (years): 15–19, 20–24, 25–29, 30–34, 35–39, 40–44, 45–49; highest educational level: no education, primary, secondary, higher; total children ever born: 1–2, 3–4, 5 + ; religion: Christianity, Islam, others/no religion; employment status: unemployed vs. employed; getting money needed for treatment/medical help: big problem vs. not a big problem; health insurance coverage: not covered vs. covered; marital status: never in union, currently in union, formerly in union; exposure to media use: no vs. yes; decision making power: low, moderate, high; wife beating justified: no vs. yes; household size: 1–4 vs. 5 + ; household wealth: poorest, poorer, middle, richer, richest; sex of household head: male vs. female; age of household head (years): < 30, 30–39, 40–49, 50–59, 60 + ; region: North Central, North East, North West, South East, South South, South West; place of residence: urban vs. rural; community ethnic diversity: mono ethnic vs. multi-ethnic.

### Analytical approach

Stata software, version 17.0, was used to analyse the data (Stata Corporation, College Station, Texas, USA). The study used a multi-stage stratified cluster sample design data, so the survey module’s (‘svy’) function was used to account for sampling design (weighting, clustering, and stratification). The percentage and chi-square test were employed in the univariate and bivariate analyses. Furthermore, Lorenz curves were used to present wealth inequalities as a plot of cumulative proportion of early maternal discharge following facility-based vaginal delivery against cumulative proportion of the population ordered by wealth status [29]. Concentration Index (CI) is positive when the Lorenz curve is below the line of equality indicating the concentration of early maternal discharge following facility-based vaginal delivery concentrates among high socioeconomic groups and vice versa. The urban-rural residence was used for stratiﬁed analyses. In the Lorenz curves, women were ranked according to ascending wealth status to estimate their position in the cumulative distribution of socioeconomic status. Conventionally, when it is applied to binary indicators, the concentration index depends on the mean of the indicator. This would impede cross-factors comparisons because there are substantial differences in means between residence. To address this issue, we used an alternative but related index introduced by Erreygers [30].

Multivariate decomposition analysis was used to decompose the percentage difference of early maternal discharge following facility-based vaginal delivery across residences and, Erreygers normalized concentration index was used to analyse the percentage contribution of factors in early maternal discharge following facility-based vaginal delivery difference across wealth quantile. A p-value less than 0.05 is considered statistically significant.

Multivariate decomposition for the non-linear model is used to quantify the contributions to group differences in average predictions from multivariate models. So, early maternal discharge following facility-based vaginal delivery deference in urban and rural areas is explained by two factors, and it could be due to the composition deference in the population (endowment) or the change in characteristics (coefficient) in the explanatory variables.

The logistic model of early maternal discharge following facility-based vaginal delivery difference in residence is given as:


$$YA-YB=F(XA\beta A)-F(XB\beta B)=\frac{F(XA\beta A)-F(XB\beta A)}{E}+\frac{F(XB\beta A)-F(XB\beta B)}{C}$$

The component *E* called the explained component, which explains the part of the differential attributable to differences in the composition of participants’ characteristics, and the *C* component, called the unexplained component, explains the part of the difference attributable to differences in effects or participants’ unexplained response.

When a variable widens the urban–rural gap in early maternal discharge, it means that the difference in the likelihood of mothers being discharged early after childbirth becomes larger between urban and rural areas. In other words, the factor increases inequality. For example, if better access to healthcare in urban areas reduces early discharge rates there but not in rural areas, the gap widens. Conversely, when a variable narrows the urban–rural gap, it reduces the disparity, bringing the rates of early discharge in both settings closer together. This could occur if an intervention, such as improved postnatal care outreach, benefits rural mothers more, thus reducing the difference. Therefore, whether a variable widens or narrows the gap reflects its influence on equity in maternal health outcomes, showing whether it promotes fairness or reinforces existing disparities between urban and rural populations.

## Results

Table 1 shows the distribution of respondents’ characteristics. Overall, mothers who had singleton birth (97.5%), average baby size at birth (53.4%), late ANC booking (71.0%), delivery in a public health facility (69.4%), Christians (62.4%), employed (76.7%), not covered by health insurance (96.4%), currently married (92.3%), exposed to mass media (80.2%), empowered: wife beating for any reason not justified (79.6%) and from male-headed household (85.3%) respectively, were more represented in the sample data. In addition, we presented the variables that showed statistical significant differences between urban and rural respondents. See Table 1 for the details.

**Table 1:** Distribution of the respondents’ characteristics.

Variable	Pooled sample (n = 8614)	Urban (n = 4446)	Rural (n = 4168)	P
**Birth type**				0.783
Singleton	8397 (97.5)	4336 (97.5)	4061 (97.4)	
Multiple	217 (2.5)	110 (2.5)	107 (2.6)	
**Birth order**				<0.001*
First	1799 (20.9)	911 (20.5)	888 (21.3)	
2^nd^/3^rd^	3191 (37.0)	1758 (39.5)	1433 (34.4)	
4^th^ and above	3624 (42.1)	1777 (40.0)	1847 (44.3)	
**Baby size at birth**				0.026*
Large	2906 (33.7)	1559 (35.1)	1347 (32.3)	
Average	4603 (53.4)	2328 (52.4)	2275 (54.6)	
Small	1105 (12.8)	559 (12.6)	546 (13.1)	
**Child sex**				0.861
Male	4489 (52.1)	2321 (52.2)	2168 (52.0)	
Female	4125 (47.9)	2125 (47.8)	2000 (48.0)	
**ANC booking**				0.176
Early	2392 (29.0)	1235 (28.4)	1157 (29.7)	
Late	5857 (71.0)	3120 (71.6)	2737 (70.3)	
**ANC visits**				<0.001*
None	357 (4.1)	86 (1.9)	271 (6.5)	
1-3	1088 (12.6)	460 (10.3)	628 (15.1)	
4-7	4010 (46.6)	1954 (44.0)	2056 (49.3)	
8+	3159 (36.7)	1946 (43.8)	1213 (29.1)	
**Place of delivery**				<0.001*
Public health facility	5981 (69.4)	2867 (64.5)	3114 (74.7)	
Private health facility	2633 (30.6)	1579 (35.5)	1054 (25.3)	
**Age (years)**				<0.001*
15-19	342 (4.0)	111 (2.5)	231 (5.5)	
20-24	1497 (17.4)	639 (14.4)	858 (20.6)	
25-29	2287 (26.6)	1209 (27.2)	1078 (25.9)	
30-34	2020 (23.4)	1152 (25.9)	868 (20.8)	
35-39	1538 (17.8)	857 (19.3)	681 (16.3)	
40-44	684 (7.9)	362 (8.1)	322 (7.7)	
45-49	246 (2.9)	116 (2.6)	130 (3.1)	
**Highest educational level**				<0.001*
No education	1425 (16.5)	431 (9.7)	994 (23.8)	
Primary	1467 (17.0)	605 (13.6)	862 (20.7)	
Secondary	4391 (51.0)	2452 (55.2)	1939 (46.5)	
Higher	1331 (15.5)	958 (21.5)	373 (9.0)	
**Total children ever born**				<0.001*
1-2	3489 (40.5)	1819 (40.9)	1670 (40.1)	
3-4	2687 (31.2)	1490 (33.5)	1197 (28.7)	
5+	2438 (28.3)	1137 (25.6)	1301 (31.2)	
**Religion**				<0.006*
Christianity	5374 (62.4)	2741 (61.7)	2633 (63.2)	
Islam	3193 (37.1)	1690 (38.0)	1503 (36.1)	
Others/no religion	47 (0.5)	15 (0.3)	32 (0.8)	
**Employment status**				0.223
Unemployed	2007 (23.3)	1012 (22.8)	995 (23.9)	
Employed	6607 (76.7)	3434 (77.2)	3173 (76.1)	
**Getting money needed for treatment/medical help**				<0.001*
Big problem	3638 (42.2)	1552 (34.9)	2086 (50.1)	
Not a big problem	4976 (57.8)	2894 (65.1)	2082 (49.9)	
**Health insurance coverage**				<0.001*
Not covered	8306 (96.4)	4219 (94.9)	4087 (98.1)	
Covered	308 (3.6)	227 (5.1)	81 (1.9)	
**Marital status**				0.049*
Never in union	314 (3.6)	143 (3.2)	171 (4.1)	
Currently in union	7951 (92.3)	4112 (92.5)	3839 (92.1)	
Formerly in union	349 (4.1)	191 (4.3)	158 (3.8)	
**Exposure to media use**				<0.001*
No	1704 (19.8)	450 (10.1)	1254 (30.1)	
Yes	6910 (80.2)	3996 (89.9)	2914 (69.9)	
**Decision making power**				<0.001*
Low	1798 (33.9)	842 (28.3)	956 (41.1)	
Moderate	1915 (36.2)	1143 (38.5)	772 (33.2)	
High	1584 (29.9)	985 (33.2)	599 (25.7)	
**Wife beating justified**				<0.001*
No	6853 (79.6)	3807 (85.6)	3046 (73.1)	
Yes	1761 (20.4)	639 (14.4)	1122 (26.9)	
**Household size**				<0.001*
1-4	3101 (36.0)	1720 (38.7)	1381 (33.1)	
5+	5513 (64.0)	2726 (61.3)	2787 (66.9)	
**Household wealth**				<0.001*
Poorest	904 (10.5)	643 (14.5)	261 (6.3)	
Poorer	1390 (16.1)	905 (20.4)	485 (11.6)	
Middle	1740 (20.2)	1023 (23.0)	717 (17.2)	
Richer	2137 (24.8)	985 (22.1)	1152 (27.6)	
Richest	2443 (28.4)	890 (20.0)	1553 (37.3)	
**Sex of household head**				<0.001*
Male	7350 (85.3)	3736 (84.0)	3614 (86.7)	
Female	1264 (14.7)	710 (16.0)	554 (13.3)	
**Age of household head (years)**				<0.001*
<30	997 (11.6)	428 (9.6)	569 (13.6)	
30-39	3209 (37.2)	1704 (38.3)	1505 (36.1)	
40-49	2380 (27.6)	1314 (29.5)	1066 (25.6)	
50-59	1039 (12.1)	518 (11.6)	521 (12.5)	
60+	989 (11.5)	482 (10.8)	507 (12.2)	
**Region**				<0.001*
North Central	1896 (22.0)	695 (15.6)	1201 (28.8)	
North East	1105 (12.8)	406 (9.1)	699 (16.8)	
North West	940 (10.9)	468 (10.5)	472 (11.3)	
South East	1723 (20.0)	1082 (24.3)	641 (15.4)	
South South	1019 (11.8)	441 (9.9)	578 (13.9)	
South West	1931 (22.4)	1354 (30.4)	577 (13.8)	
**Community ethnic diversity**				<0.001*
Mono ethnic	4354 (50.6)	1996 (44.9)	2358 (56.6)	
Multi ethnic	4260 (49.4)	2450 (55.1)	1810 (43.3)	

* significant at *P* < 0.05.

**Fig 1:**
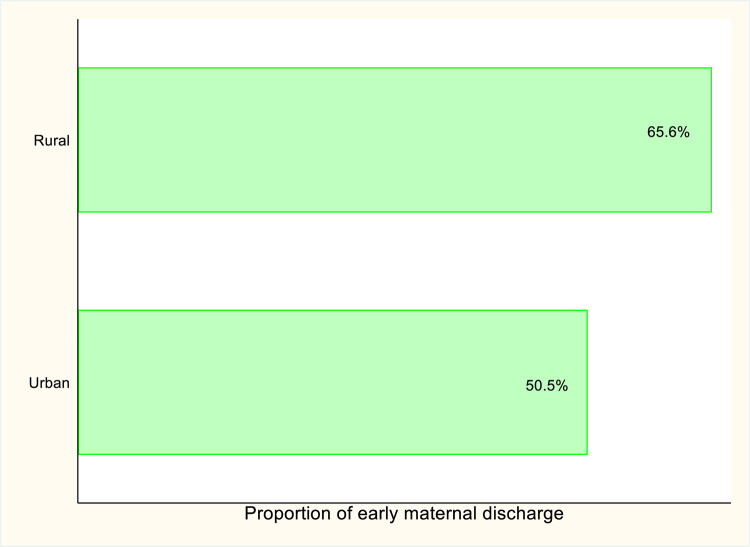
Percentage of early maternal discharge following vaginal delivery by urban-rural differences.

Fig 1 shows the percentage distribution of early maternal discharge across urban-rural residence. Approximately 65.6% of rural women and 50.5% of urban dwellers respectively, reported early maternal discharge following vaginal delivery in Nigeria.

**Fig 2:**
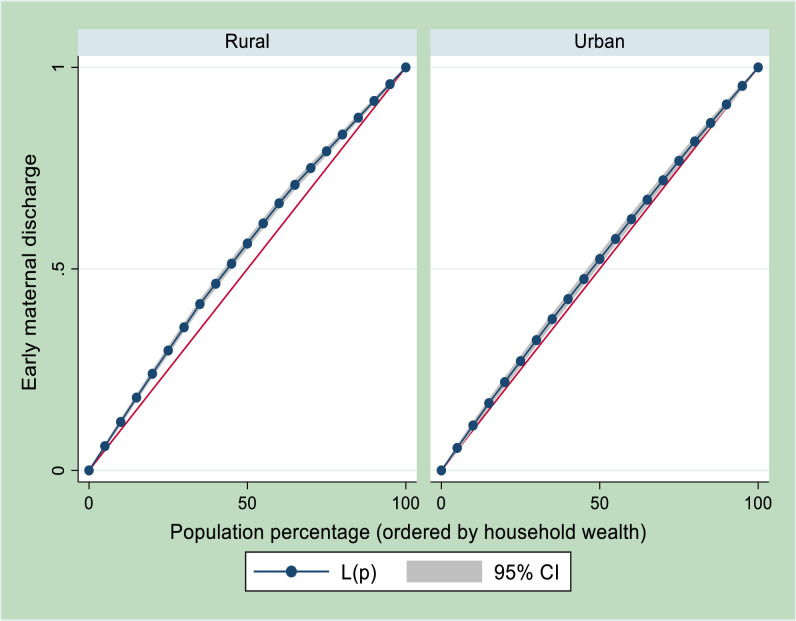
Urban-rural differential in socioeconomic inequalities of early maternal discharge following vaginal delivery.

Fig 2 presents the Lorenz curves for urban-rural inequalities by wealth status. The result shows pro-poor distribution of early maternal discharge following vaginal delivery among urban and rural women. The concentration index for rural residence was -0.0817 (SE = 0.0063; p < 0.001) and was -0.0346 (SE = 0.0083; p < 0.001) for urban residence. The z-stat was -4.53 indicating a statistical significant difference (p < 0.001) between urban and rural residence in Nigeria.

Table 2 show the estimated percentage distribution of early maternal discharge following vaginal delivery by urban-rural residence. The results consistently showed that across the levels of women’s characteristics, the rural women had higher proportion of early maternal discharge in Nigeria.

**Table 2:** Percentage change (∆%) of early maternal discharge following vaginal delivery.

Variable	Urban (%)	Rural (%)	Percentage point difference (∆%)
**Birth type**			
Singleton	52.5	65.3	-12.8
Multiple	37.7	49.5	-11.8
**Birth order**			
First	48.3	62.5	-14.2
2^nd^/3^rd^	50.3	64.5	-14.2
4^th^ and above	55.9	66.3	-10.4
**Baby size at birth**			
Large	56.9	69.6	-12.7
Average	49.0	62.4	-13.4
Small	52.0	63.5	-11.5
**Child sex**			
Male	52.0	65.7	-13.7
Female	52.4	63.9	-11.5
**ANC booking**			
Early	46.8	62.8	-16.0
Late	54.3	64.5	-10.2
**ANC visits**			
None	52.9	79.0	-26.1
1-3	63.0	75.0	-12.0
4-7	55.2	65.9	-10.7
8+	46.5	55.1	-8.6
**Place of delivery**			
Public health facility	57.1	68.3	-11.2
Private health facility	58.0	52.8	5.2
**Age (years)**			
15-19	45.9	66.7	-20.8
20-24	54.3	70.4	-16.1
25-29	53.3	65.5	-12.2
30-34	51.0	62.4	-11.4
35-39	53.6	61.6	-8.0
40-44	46.5	64.1	-17.6
45-49	53.6	54.8	-1.2
**Highest educational level**			
No education	69.3	77.1	-7.8
Primary	56.1	65.2	-9.1
Secondary	48.5	59.9	-11.4
Higher	51.2	56.9	-5.7
**Total children ever born**			
1-2	49.0	63.8	-14.8
3-4	53.7	62.6	-8.9
5+	55.1	68.3	-13.2
**Religion**			
Christianity	42.3	58.4	-16.1
Islam	67.9	76.0	-8.1
Others/no religion	40.0	43.3	-3.3
**Employment status**			
Unemployed	58.2	67.5	-9.3
Employed	50.3	64.0	-13.7
**Getting money needed for treatment/medical help**			
Big problem	51.2	66.8	-15.6
Not a big problem	52.7	63.0	-10.3
**Health insurance coverage**			
Not covered	52.0	64.9	-12.9
Covered	54.9	60.8	-5.9
**Marital status**			
Never in union	32.8	55.9	-23.1
Currently in union	52.7	65.4	-12.7
Formerly in union	54.3	61.7	-7.4
**Exposure to media use**			
No	61.2	72.2	-11.0
Yes	51.1	61.7	-10.6
**Decision making power**			
Low	52.9	65.3	-12.4
Moderate	52.6	63.6	-11.0
High	45.6	52.4	-6.8
**Wife beating justified**			
No	50.3	62.5	-12.2
Yes	63.0	71.2	-8.2
**Household size**			
1-4	48.5	61.2	-12.7
5+	54.5	66.6	-12.1
**Household wealth**			
Poorest	58.2	78.0	-19.8
Poorer	54.4	78.3	-23.9
Middle	51.9	74.8	-22.9
Richer	50.3	64.7	-14.4
Richest	47.9	54.0	-6.1
**Sex of household head**			
Male	53.6	66.2	-12.6
Female	44.5	56.1	-11.6
**Age of household head (years)**			
<30	51.3	69.2	-17.9
30-39	52.5	66.6	-14.1
40-49	52.7	62.9	-10.2
50-59	52.6	65.3	-12.7
60+	49.9	58.6	-8.7
**Region**			
North Central	72.5	77.6	-5.1
North East	80.3	87.4	-7.1
North West	77.5	78.6	-1.1
South East	32.7	39.2	-6.5
South South	36.7	38.2	-1.5
South West	44.1	50.1	-6.0
**Community ethnic diversity**			
Mono ethnic	46.0	64.8	-18.8
Multi ethnic	57.2	65.0	-7.8

Table 3 presents the results of the decomposition analysis at the aggregate level for the changes due to differences in compositional factors or characteristics (E) and those due to differences in coefficients or unexplained (C), as well as the contribution of each predictor to the overall component of E and C. Results indicated that, about 17.6% of early maternal discharge gap was explained by the differences in distributions of characteristics (endowments) between urban and rural residence, while 82.4% of early maternal discharge gap was due to the differences in the distribution of unexplained factors urban and rural residence. In the urban-rural decomposition analysis, a difference in characteristics (E) value of -0.56%, 10.38%, -6.95%, -15.21%, -7.53%, -4.73%, 4.11%, 10.17%, 26.41%, -18.93% and 55.36% for women with multiple births, delivery at private hospitals, secondary education, higher education, moderate decision making power, high decision making power, 5 + household size, North East, South east, South South and South West respectively, indicates that the distribution of women’s variables widened or narrowed the urban-rural gap in early maternal discharge, depending on whether the values take a positive of negative sign.

**Table 3:** Factors contributing to urban-rural inequality in early maternal discharge following vaginal delivery.

Variable	Endowments (Explained) (E)		Coefficients (Unexplained) (C)	
	β (SE)	Percentage contribution (Pct)	β (SE)	Percentage contribution (Pct)
**Overall**	0.0172 (0.0157)	17.6	0.0807 (0.0206)	82.4
**Birth type**				
Singleton	Ref		Ref	
Multiple	-0.0006 (0.0002)*	-0.56	0.0011 (0.0020)	1.10
**Birth order**				
First	Ref		Ref	
2^nd^/3^rd^	-0.0024 (0.0021)	-2.47	0.0195 (0.0189)	19.92
4^th^ and above	0.0026 (0.0035)	2.70	-0.0025 ()0.0286	-2.60
**Baby size at birth**				
Large	Ref		Ref	
Average	-0.0006 (0.0001)	-0.66	0.0141 (0.0152)	14.42
Small	-0.0001 (0.0001)	-0.15	0.0006 (0.0053)	0.58
**Child sex**				
Male	Ref		Ref	
Female	-0.0001 (0.0003)	-0.11	0.0020 (0.0128)	2.04
**ANC booking**				
Early	Ref		Ref	
Late	0.0001 (0.0002)	0.11	-0.0075 (0.0213)	14.42
**ANC visits**				
1-3	Ref		Ref	
4-7	-0.0015 (0.0033)	-1.55	0.0088 (0.0202)	9.02
8+	-0.0109 (0.0061)	-11.11	0.0290 (0.0247)	29.63
**Place of delivery**				
Public health facility	Ref		Ref	
Private health facility	0.0102 (0.0032)*	10.38	-0.0177 (0.0112)	-18.05
**Age (years)**				
15-19	Ref		Ref	
20-24	0.0012 (0.0031)	1.18	0.0062 (0.0117)	6.36
25-29	0.0002 (0.0007)	0.19	0.0006 (0.0279)	0.57
30-34	-0.0005 (0.0040)	-0.47	0.0001 (0.0317)	0.07
35-39	-0.0009 (0.0022)	-0.93	0.0032 (0.0243)	3.29
40-44	-0.0001 (0.0008)	-0.09	0.0049 (0.0114)	4.98
45-49	-0.0007 (0.0041)	-0.72	-0.0017 (0.0034)	-1.77
**Highest educational level**				
No education	Ref		Ref	
Primary	0.0029 (0.0028)	2.95	-0.0025 (0.0078)	-2.56
Secondary	-0.0068 (0.0035)*	-6.95	0.0120 (0.0285)	12.25
Higher	-0.0149 (0.0073)*	-15.21	0.0142 (0.0152)	14.46
**Total children ever born**				
1-2	Ref		Ref	
3-4	0.0012 (0.0013)	1.22	-0.0059 (0.0163)	-6.00
5+	-0.0007 (0.0042)	-0.73	0.0167 (0.0177)	17.06
**Religion**				
Christianity	Ref		Ref	
Islam	0.0001 (0.0006)	0.09	-0.0142 (0.0135)	-14.46
Others/no religion	-0.0005 (0.0006)	-0.53	-0.0007 (0.0006)	-0.73
**Employment status**				
Unemployed	Ref		Ref	
Employed	0.0001 (0.0006)	0.13	-0.0019 (0.0731)	-1.97
**Getting money needed for treatment/medical help**				
Big problem	Ref		Ref	
Not a big problem	-0.0007 (0.0023)	-0.69	0.0244 (0.0185)	24.90
**Health insurance coverage**				
Not covered	Ref		Ref	
Covered	-0.0017	-1.70	0.0059 (0.0042)	6.02
**Exposure to media use**				
No	Ref		Ref	
Yes	-0.0034 (0.0054)	-3.43	-0.0149 (0.0409)	-15.17
**Decision making power**				
Low	Ref		Ref	
Moderate	-0.0074 (0.0020)*	-7.53	0.0144 (0.0130)	14.71
High	-0.0046 (0.0023)*	-4.73	-0.0086 (0.0122)	-8.74
**Wife beating justified**				
No	Ref		Ref	
Yes	-0.0019 (0.0024)	-1.98	-0.0109 (0.0048)*	-11.08
**Household size**				
1-4	Ref		Ref	
5+	0.0040 (0.0018)*	4.11	0.0055 (0.0222)	5.59
**Household wealth**				
Poorest	Ref		Ref	
Poorer	-0.0088 (0.0081)	-9.02	0.0283 (0.0145)	28.84
Middle	-0.0005 (0.0048)	-0.51	0.0252 (0.0152)	25.75
Richer	-0.0013 (0.0035)	-1.27	0.0167 (0.0151)	17.02
Richest	-0.0126 (0.0157)	-12.82	0.0171 (0.0146)	17.40
**Sex of household head**				
Male	Ref		Ref	
Female	0.0002 (0.0003)	0.25	-0.0043 (0.0053)	-4.40
**Age of household head (years)**				
<30	Ref		Ref	
30-39	0.0013 (0.0009)	1.36	-0.0248 (0.0201)	-25.31
40-49	0.0025 (0.0018)	2.50	-0.0199 (0.0186)	-20.31
50-59	-0.0001 (0.0002)	-0.11	-0.0032 (0.0075)	-3.22
60+	-0.0005 (0.0010)	-0.48	0.0034 (0.0062)	3.47
**Region**				
North Central	Ref		Ref	
North East	0.0100 (0.0031)*	10.17	0.0029 (0.0043)	2.96
North West	0.0034 (0.0019)	3.47	-0.0025 (0.0052)	-2.51
South East	0.0259 (0.0044)*	26.41	0.0017 (0.0125)	1.75
South South	-0.0185 (0.0032)*	-18.93	-0.0109 (0.0053)*	-11.08
South West	0.0542 (0.0104)*	55.36	-0.0257 (0.0149)	-26.26
**Community ethnic diversity**				
Mono ethnic	Ref		Ref	
Multi ethnic	0.0005 (0.0018)	0.48	-0.0062 (0.0152)	-6.34

β., Decomposition coefficients; Pct., Percentage contribution of each variable category to early maternal discharge following vaginal delivery; Ref., Reference; SE., standard error; * significant at *P* < 0.05.

## Discussion

In Nigeria, early maternal discharge following vaginal delivery was found to be more prevalent in rural areas due to limited health facility access, workforce shortages, and sociocultural norms. Early maternal discharge in rural Nigeria mirrors trends across Africa [25,31], which could be driven by poor health access, workforce shortages, and sociocultural factors, posing risks to maternal and newborn health. In addition, there was a pro-poor distribution of early maternal discharge among Nigerian women with poor women having higher distribution of early maternal discharge following facility-based delivery, both in rural and urban residence. Based on our findings, the distribution of women having multiple births narrowed the urban-rural gap in early maternal discharge. Multiple births are typically classified as higher-risk deliveries, prompting healthcare providers to recommend longer postpartum observation, regardless of location [32]. This increased clinical attention reduces the likelihood of early maternal discharge among rural and urban women with multiple births [25]. Therefore, multiple births in rural or urban areas can lead to more extended hospital stays among these women, mitigating the disparity in early maternal discharge rates by place of residence. This trend highlights how clinical risk factors can counterbalance structural inequalities in maternal healthcare access.

The findings from this study show that delivery in private hospitals widened urban-rural gap in early maternal discharge following vaginal delivery. There is disproportionately more private health facilities in urban than rural areas, contributing to a widening urban-rural gap in early maternal discharge following vaginal delivery. Private health facilities, which are more concentrated in urban settings, often operate under profit-driven models and resource optimization pressures [33], which may result in shorter postpartum stays to accommodate more patients for the hospital and reduce catastrophic out-of-pocket expenditures for women. Consequently, urban women who deliver in private hospitals may be more likely to experience early maternal discharge compared to their rural counterparts, who predominantly use public or primary healthcare centers where discharge protocols may be more conservative due to fewer deliveries and lower patient turnover [34]. Moreover, rural areas face limited access to private facilities due to cost, distance, and availability [35]. The urban dominance of private hospital deliveries therefore could skew early maternal discharge prevalence, exacerbating the urban-rural disparity in postpartum care.

Another key result from this study shows that higher education narrowed urban-rural inequality in early maternal discharge following vaginal delivery. Women with higher education are more empowered to make decision especially regarding their health care. Therefore, whether and educated woman delivers in rural or urban areas, she will be able to make proper health care decision for herself and child whether there is faster patient turnover, higher delivery volumes, and non-adherence to institutional protocols by health workers [36]. However, uneven distribution in educational attainment between urban-rural women may lead to disparity in health care services uptake. In addition, attending ANC could also contribute to women’ knowledge pertaining to perinatal period.

Furthermore, women’s decision-making power narrowed urban-rural gap in early maternal discharge following vaginal delivery. Good decision-making power by women irrespective of their place of residence, may help narrow the urban-rural gap in early maternal discharge following vaginal delivery. Women with greater autonomy are more likely to influence their health care decisions [37,38], including the timing of discharge, based on personal preferences and perceived readiness rather than facility-driven protocols or inadequacy in skilled birth attendants, infrastructure amongst others. In rural areas, where early discharge is prevalent due to limited postnatal support, cultural norms, and shortage in staff, the empowered women may advocate for optimal postnatal care including the duration of stay before discharge, aligning with their urban counterparts who are empowered. Women who are empowered by wealth, education, socioeconomic status can resist premature discharge if they perceive health risks, despite systemic pressure for fast turnover in crowded facilities. This self-advocacy across settings reduces extremes in discharge timing. Therefore, increased distribution of women with high decision-making power fosters more balanced maternal care experiences, leading to a convergence in early maternal discharge rates between urban and rural populations.

We found large household size to widen urban-rural gap in early maternal discharge following facility-based vaginal delivery. Large household sizes are more commonly found in rural areas, and this distribution may widen the urban-rural gap in early maternal discharge following vaginal delivery [39]. In rural settings, large households often provide immediate postpartum support, allowing women to leave health facilities earlier with confidence in home-based care. In addition, cultural norms in rural areas may encourage early discharge to resume domestic responsibilities, especially in extended families. However, urban women, who typically belong to smaller nuclear households, may rely more on institutional care for postpartum support and prefer longer facility stays. Urban health providers may also retain women longer in the absence of sufficient home support systems. As a result, rural women with large household support may be more frequently discharged early, while urban women from smaller households may remain longer, exacerbating the urban-rural disparity in early maternal discharge. Thus, household size distribution significantly influences the pattern of postpartum discharge practices.

Living in the South South geographical region of Nigeria narrowed urban-rural inequality in early maternal discharge following facility-based vaginal delivery. The South-South may have a more balanced urban-rural distribution or characteristics of women giving birth, with relatively homogeneous access to healthcare services across settings. The cultural norms and health infrastructure in the South South promote consistent postpartum care regardless of residence, resulting in similar discharge patterns between urban and rural women, as found in this study. Thus, while other regions (North East, South East and South West) widened the urban-rural gap, the South South region contributes to narrowing it. The North-East, South-East, and South-West regions of Nigeria contribute to a widened urban-rural gap in early maternal discharge due to differing health service access and utilization patterns. Urban areas in these regions often have higher delivery rates in overcrowded facilities, which may be prompting providers to discharge women early to manage patient load. These regional dynamics may deepen urban-rural disparity in postpartum care outcomes.

The South South region of Nigeria may exhibit cultural norms that promote favourable postpartum outcomes among women due to its strong emphasis on communal care, traditional confinement practices, and maternal support systems. In many ethnic groups within the region, such as the Urhobo, Ijaw, and Efik, childbirth is viewed as a collective event, and new mothers receive intensive support from extended family members during the postpartum period. Cultural practices such as “omugwo” or “uwe” involve mothers or elderly female relatives caring for the new mother for several weeks after delivery, ensuring rest, balanced nutrition, and emotional support. These traditions help prevent early maternal discharge and complications by allowing recovery under supervision. Also, higher literacy and female empowerment levels in parts of the South South, often associated with oil-related economic development, enhance women’s decision-making power regarding healthcare utilization. The region’s relatively higher access to urban healthcare facilities also facilitates appropriate postnatal follow-up. Compared with some northern regions where early discharge or limited facility delivery may be influenced by sociocultural or religious norms restricting women’s autonomy, South South customs prioritize maternal wellbeing and social support. Collectively, these cultural, educational, and socioeconomic factors contribute to improved postpartum health outcomes among women in the South South region.

## Strengths and limitations

This study’s findings are plausible because it used nationally representative data from the 2018 NDHS. By focusing on urban-rural inequality in early maternal discharge, policymakers can improve the delivery of health information, counselling, and programmes to address the observed gap in early maternal delivery. However, early maternal discharge was evaluated using self-reported data, which may introduce recall or social desirability bias. In addition, the assets-based wealth index, which is employed as a stand-in for household economic status, might not always yield accurate results when compared to reliable direct measurements of income and expenditure. This study utilized secondary data and was unable to measure epidural analgesia uptake among the respondents. In addition, birth complications could influence the length of hospital stay, but this was not captured in the secondary data.

## Conclusion

The findings of this study make significant contributions to the existing body of literature on maternal health in Nigeria by highlighting the persistence and determinants of inequities in early maternal discharge between urban and rural populations. The evidence that early discharge is more prevalent among rural women than urban women and that the practice follows a pro-poor distribution underscores ongoing challenges in equitable access to quality postnatal care. The negative concentration indices indicate that poorer women are disproportionately affected, reinforcing the need for poverty-sensitive interventions. The study advances understanding by identifying specific factors that narrow or widen the gap, such as education, decision-making power, and facility type, offering insights into context-specific policy actions. By pinpointing regions and social conditions associated with disparities, the study provides an empirical basis for designing targeted strategies to improve maternal care equity, strengthen postnatal health systems, and guide national efforts toward achieving Sustainable Development Goal 3 on reducing maternal morbidity and mortality in Nigeria.
